# Prognostic factors of invasive fungal infections in pediatric intensive care units and changes in treatment outcomes before and after the COVID-19 pandemic: a multicenter retrospective study

**DOI:** 10.3389/fmicb.2025.1605960

**Published:** 2025-05-30

**Authors:** Yuhui Du, Li Huang, Xiulan Lu, Jun Su, Lidan Cui, Qunqun Zhang, Liming He, Tian Li, Ling Gong, Guoping Lu, Yibing Cheng

**Affiliations:** ^1^Department of Pediatric Intensive Care Unit, Children′s Hospital Affiliated to Zhengzhou University (Henan Children’s Hospital), Zhengzhou, China; ^2^Department of Pediatric Intensive Care Unit, Guangzhou Women and Children's Medical Center, The Affiliated Hospital of Guangzhou Medical University, Guangzhou, China; ^3^Department of Pediatric Intensive Care Unit, The Affiliated Children's Hospital of Xiangya School of Medicine, Central South University (Hunan Children's Hospital), Changsha, China; ^4^Department of Pediatric Intensive Care Unit, Children’s Hospital of Fudan University, Shanghai, China

**Keywords:** invasive fungal infection, critically ill children, prognosis, COVID-19 pandemic, co-infection

## Abstract

**Objective:**

To investigate the prognostic factors influencing the outcomes of children with invasive fungal infection in pediatric intensive care unit (PICU) and explore the effects of changes in clinical characteristics before and after the COVID-19 pandemic on treatment outcomes.

**Methods:**

In total, 665 children with invasive fungal infections from the PICUs of four hospitals in China were retrospectively enrolled from January 2017 to December 2021. These children were categorized into an effective treatment group (336 cases) and a failure group (329 cases, including treatment abandonment and death) based on prognosis. Clinical data were compared between the pre-pandemic period (2017–2019, 421 cases) and the post-pandemic period (2020–2021, 244 cases). Univariate and multivariate logistic regression analyses were used to identify the factors related to prognosis.

**Results:**

Prognostic factors: Independent risk factors for treatment failure included agranulocytosis (OR = 3.389, 95% CI 1.518–6.287), hematological malignancies (OR = 3.050, 95% CI 1.544–5.986), blood transfusion (OR = 2.239, 95% CI 1.456–3.442), invasive mechanical ventilation (OR = 1.938, 95% CI 1.158–3.243), and indwelling urinary catheter (OR = 1.750, 95% CI 1.048–2.924). The independent protective factor was identified fungal pathogens (OR = 0.588, 95% CI 0.362–0.956). Pre- and post-pandemic comparisons revealed that the co-infection rate decreased after pandemic (77.9% vs. 70.5%, *p* < 0.05), the proportion of identified fungal pathogens increased (60.6% vs. 68.0%, *p* = 0.054), whereas, the treatment failure rate was significantly higher (45.8% vs. 55.7%, *p* < 0.05). Changes in fungal species after the pandemic: The proportions of *Candida* and *Aspergillus* had decreased (89.0% vs. 75.9%, *p* < 0.05; 8.2% vs. 6.0%, *p* > 0.05), whereas those of *Pneumocystis jirovecii* and other fungal species had increased significantly (0.4% vs. 7.8, 2.4% vs. 10.2%, all *p* < 0.05).

**Conclusion:**

Agranulocytosis, hematologic malignancies, invasive mechanical ventilation, indwelling urinary catheter and blood transfusion are independent risk factors for adverse outcomes with invasive fungal infections in PICU, and identifying the pathogen can improve outcomes. Post-pandemic changes in fungal species and cumulative risk factors may have offset the potential benefits of reduced co-infection rates, leading to increased treatment failure rates. Therefore, it is necessary to optimize invasive procedure management and provide early coverage for emerging pathogens in high-risk children.

## 1 Introduction

Invasive fungal infections (IFIs) are caused by opportunistic pathogens, and the risk of infection is determined by the interplay of host susceptibility and environmental exposure. Despite advancements in medical technology, the morbidity and mortality rates associated with IFIs remain high to date. Fungal infections account for 20% of all the microbiologically confirmed infections in intensive care units (ICUs) (Vincent et al., 2009). Among critically ill adults, IFI-associated mortality ranges from 40 to 60%, whereas pediatric invasive aspergillosis (IA) is associated with an even higher mortality rate of up to 70%, making IFI a major threat to survival for PICU patients (Garnacho-Montero et al., 2018; Hon et al., 2024; Pardo et al., 2019; Reizine et al., 2024). Co-infections, particularly with multiple pathogens, may result in worse clinical outcomes because of compounded immunosuppression (Zhao et al., 2021). The national containment measures adopted during and after the COVID-19 pandemic significantly reduced the overall pathogen detection and co-infection rates (Achangwa et al., 2022; Sauteur et al., 2022), although systematic studies on how respiratory virus epidemiology impacts IFI prognosis or pathogen distribution in children are lacking even today. In this regard, the present research was designed as a retrospective study to analyze the clinical and microbiological data of IFI patients admitted to the PICUs across four Chinese hospitals between January 2017 and December 2021. The study, through a comparison of the pre-pandemic and post-pandemic periods, aimed to identify the prognostic factors for poor outcomes and provide evidence-based guidance for early clinical intervention in these patients.

## 2 Methods

### 2.1 Study subjects

Case screening: This multicenter retrospective study reviewed the medical records of the pediatric patients diagnosed with IFI admitted to the PICUs of the Children’s Hospital Affiliated to with Zhengzhou University, Shanghai Fudan University Children’s Hospital, Hunan Children’s Hospital, and Guangzhou Women and Children’s Medical Center between 1 January 2017 and 31 December 2021. When this study ended, China was still under epidemic control measures, and it was confirmed that none of the included PICUs had any cases of COVID-19 infection. Patients meeting the following criteria were enrolled in this study: (1) aged 1 month to 18 years; (2) underwent pathogenic microbiological testing; (3) met the diagnostic criteria for IFI (Donnelly et al., 2020) (including possible, probable, and proven cases). Patients with incomplete data were excluded. In total, 665 cases were included in the final analysis.

### 2.2 Collection of clinical data and study grouping

Clinical data of the included children were retrieved from the electronic medical record systems of the respective hospital. (1) General information: Basic demographics, underlying diseases, and routine laboratory tests. (2) Host risk factors: presence of invasive mechanical ventilation (IMV), agranulocytosis, immunosuppressive therapy, parenteral nutrition, malnutrition, indwelling intravascular catheters, blood purification, extracorporeal membrane oxygenation (ECMO), surgical procedures, blood transfusions, and indwelling urinary catheters. (3) Co-infections at the time of IFI diagnosis: Pathogen data including fungal, bacterial, and viral infections. (4) Prognostic indicators: length of stay in the PICU (PICU LoS), length of stay in the hospital (hospital LoS), and treatment outcomes: effective (the remission or disappearance of the clinical manifestations related to IFI in children during hospitalization) or failure (the disease progression leading to treatment abandonment and in-hospital death).

Patients were classified into the following groups based on treatment outcomes: the treatment-effective group and the treatment-failure group. IFI patients admitted to the PICU between 1 January 2017 and 31 December 2019 were categorized into the pre-pandemic group, whereas those admitted between 1 January 2020 and 31 December 2021 were categorized into the post-pandemic group.

### 2.3 Statistical analysis

Data analysis was performed using SPSS 23.0 statistical software, and GraphPad Prism 9.5 was used for plotting graphs. Continuous data were tested for normality using the Shapiro–Wilk test. Normally distributed data were presented as the means ± standard deviations (x̄±s), whereas the non-normally distributed data were presented as medians (P_25_, P_75_). Intergroup comparisons for non-normally distributed data were conducted using the Kruskal–Wall is test. Categorical data were expressed as numbers (%), and intergroup differences were assessed using the Chi-squares test. Variables with statistical significance in the univariate analysis were included in a multivariate logistic regression model, and a forest plot was generated. A two-sided *p* < 0.05 indicated statistical significance.

## 3 Results

In total, 665 children were included in this analysis, among which 397 were males and 268 were females (median age: 18 months). Among these cases, 336 cases were placed in the effective treatment group, and 329 were placed in the failure group (including 218 cases of treatment abandonment and 111 cases of in-hospital mortality), leading to a treatment failure rate of 49.5%. Statistically significant differences (*p* < 0.05) were noted between the two groups in terms of age, weight, PICU LoS, hospital LoS, proportion of patients with hematologic malignancies, and diagnostic stratification. Compared to the patients in the effective group, those in the failure group were older and had greater weights, shorter PICU/hospitalization durations, and a greater proportion of hematologic malignancies (Table 1).

**Table 1:** Comparison of baseline and clinical characteristics between the effective treatment group and the treatment failure group.

Groups	Age (months)	Weight (kg)	Sex (Male, %)	PICU LoS/day	Hospital LoS/day	hematologic malignancies	Diagnostic stratification		
							Possible cases	Probable cases	Proven cases
Treatment-effective (*n* = 336)	12.5 (4.0,52.8)	9.0 (5.7,15.0)	212 (63.1)	20.5 (10.3,38.0)	30.0 (19.0,48.0)	19 (5.7)	80 (23.8)	166 (49.4)	90 (26.8)
Treatment-failure (*n* = 329)	28.0 (7.0,83.5)	11.0 (6.6,20.6)	185 (56.2)	16.0 (7.0,33.5)	25.0 (12.0,42.0)	73 (22.2)	116 (35.3)	102 (31.0)	111 (33.7)
*Χ*^2^/*Z*	−4.179	−3.793	3.255	−2.919	−4.303	38.120	24.019		
*p*-value	0.000	0.000	0.071	0.004	0.000	0.000	0.000		

Data are presented as the median (IQR) or frequency (%). Los, length of stay.

### 3.1 Host risk factors in IFI patients

Compared to the treatment-effective group, the treatment-failure group presented significantly higher utilization rates of IMV, agranulocytosis, immunosuppressants, intravascular catheters, blood purification, ECMO, blood transfusion, and indwelling urinary catheters (*p* < 0.05). The treatment failure group presented higher rates of parenteral nutrition and malnutrition incidence compared to the effective treatment group, along with fewer surgical interventions, although these differences lacked statistical significance (*p* > 0.05). The effective treatment group had a greater proportion of children with identified fungal pathogens (*p* < 0.05) (Table 2).

**Table 2:** Comparison of host risk factors between the effective treatment group and the treatment failure group.

Groups	IMV	Agranulocytosis	IS	PN	Malnutrition	IVC	BP	ECMO	Surgery	BT	IUC	IFP
Treatment-effective (*n* = 336)	187 (55.7)	17 (5.1)	35 (10.4)	57 (17.0)	108 (32.1)	177 (52.7)	40 (11.9)	6 (1.8)	80 (23.8)	146 (43.5)	157 (46.7)	227 (67.6)
Treatment-failure (*n* = 329)	268 (81.5)	66 (20.1)	52 (15.8)	73 (22.2)	122 (37.1)	243 (73.9)	84 (25.5)	15 (4.6)	62 (18.8)	241 (73.3)	245 (74.5)	194 (59.0)
*Χ*^2^/*Z*	51.228	34.246	4.245	2.885	1.792	32.052	20.349	4.183	2.440	60.678	53.512	5.284
*P*-value	0.000	0.000	0.039	0.089	0.181	0.000	0.000	0.041	0.118	0.000	0.000	0.022

Data are presented as frequency (%). IMV, invasive mechanical ventilation; IS, immunosuppressants; PN, parenteral nutrition; IVC, intravascular catheter; BP, blood purification; ECMO, extracorporeal membrane oxygenation; BT, blood transfusion; IUC, indwelling urinary catheter; IFP, identified fungal pathogen.

### 3.2 Distribution of fungal species, treatment outcomes, and co-infections with common pathogens

**Figure 1:**
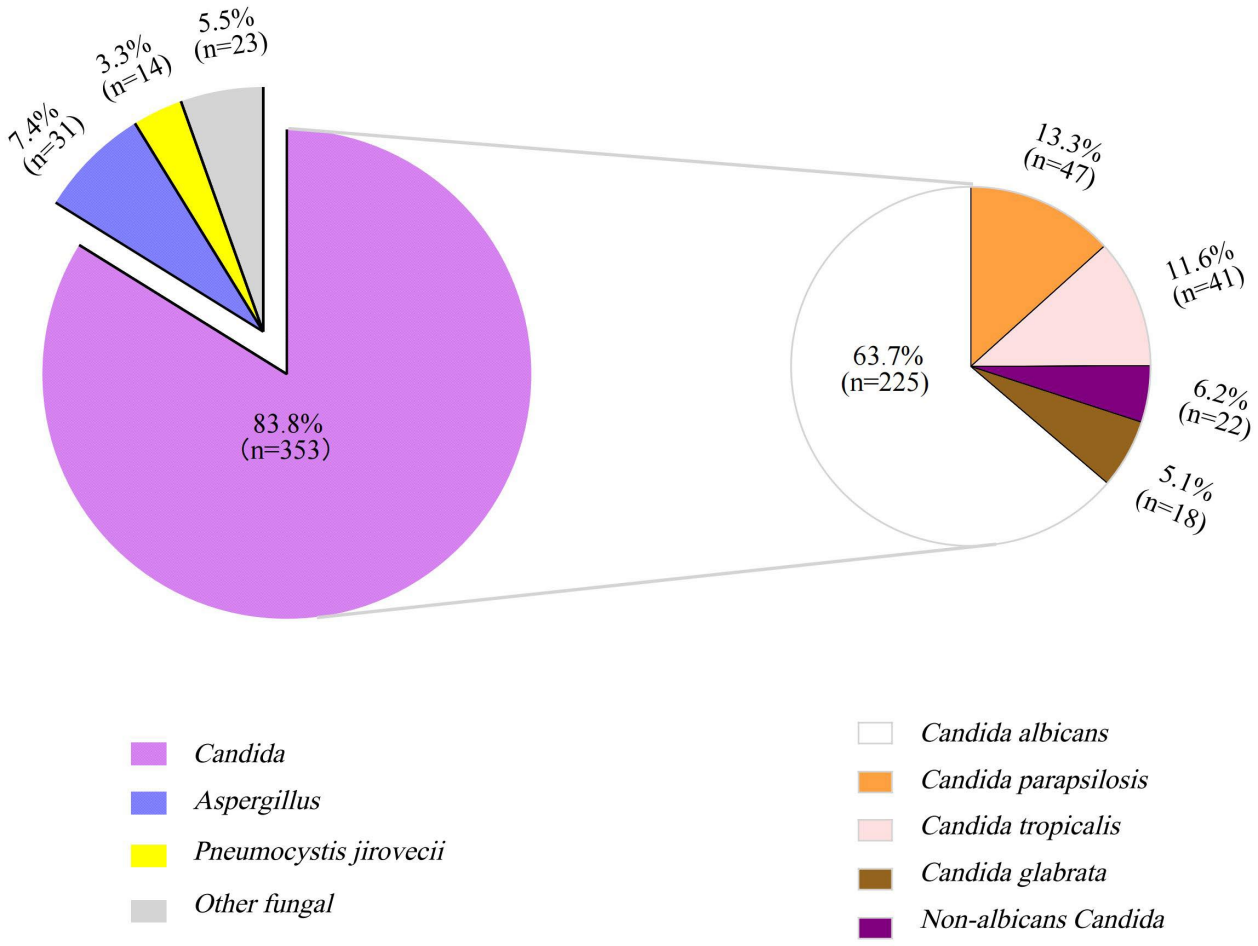
Proportion of different fungal species in children with invasive fungal infections in PICU (%). The larger purple pie chart represents *Candida* infections, the smaller one illustrates the species-specific distribution of *Candida* infections.

**Figure 2:**
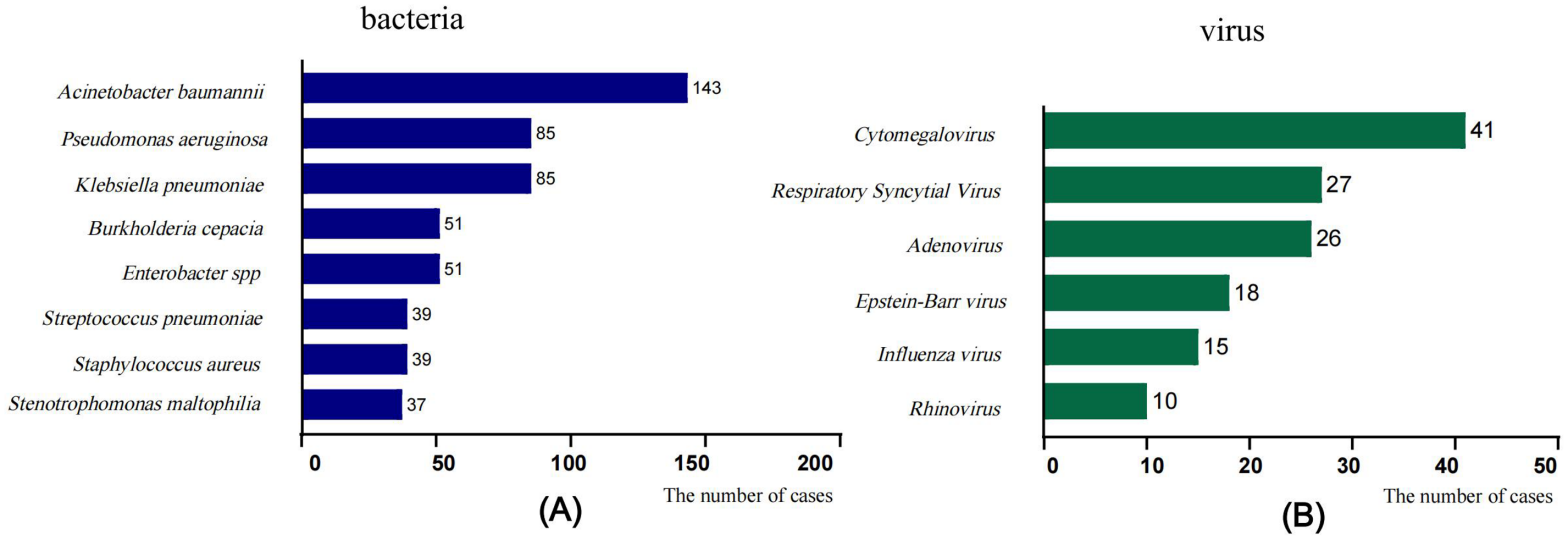
Fungal co-infections with common pathogens. **(A)** Fungal-bacterial co-infections, **(B)** Fungal-viral co-infections. The *x*-axis displays the number of isolates in which the corresponding pathogen species were found.

The pathogens of IFI in the PICU were predominantly *Candida* species, with *Candida albicans* being the most common one, followed by *Candida parapsilosis*, *Candida tropicalis*, and *Candida glabrata* (Figure 1). Treatment failure rates varied among different fungal species, with the highest treatment failure rate of 82.6% noted for the other fungal species category (including Mucor, *Talaromyces marneffei*, *Geotrichum capitatum*, *Cryptococcus*, etc.), followed by *Aspergillus* species (71.2%) (Table 3). The pure fungal infection rate was 24.8%, whereas co-infections accounted for 75.2%. Among co-infections, fungal-bacterial co-infections accounted for 65.9%, with *Acinetobacter baumannii* being the most common bacterial pathogen causing co-infection. Fungal-viral co-infections accounted for 22.1%, and cytomegalovirus was the most frequently detected virus (Figure 2 and Table 4).

**Table 3:** Treatment outcomes.

Fungal species	*Candida*	*Aspergillus*	*Pneumocystis jirovecii*	Other fungi
Treatment failure rates (%)	144/353 (40.8)	23/31 (71.2)	8/14 (57.1)	19/23 (82.6)

**Table 4:** Co-infections with common pathogens.

Classification	Pure fungal infection	Fungal-bacterial co-infections	Fungal-viral co-infections
The proportion (%)	165/665 (24.8)	438/665 (65.9)	147/665 (22.1)

### 3.3 Forest plot of the results from multivariate logistic regression analysis for pediatric patients with IFI

**Figure 3:**
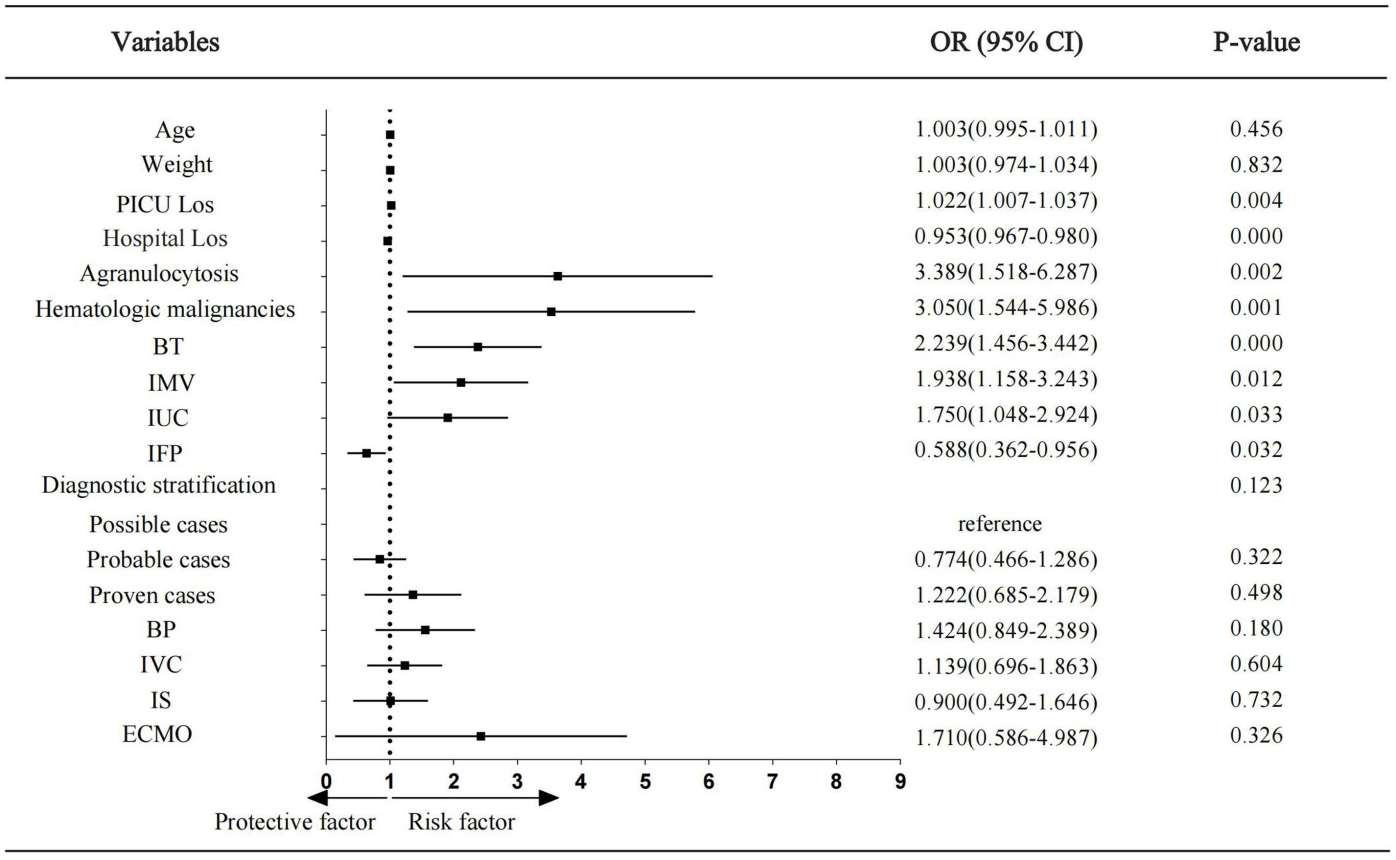
Forest plot showing odds ratios of multivariate logistic regression analysis. Los, length of stay; BT, blood transfusion; IMV, invasive mechanical ventilation; IUC, indwelling urinary cathete; IFP, identified fungal pathogen; BP, blood purification; IVC, intravascular catheter; IS, immunosuppressants; ECMO, extra corporeal membrane oxygenation; OR, odds ratio; CI, confidence interval.

The factors with *p* < 0.05 identified in the univariate analysis results, presented in Tables 1, 2, were utilized to establish the multivariate logistic regression model to explore their impact on treatment failure in children with IFI in PICU. The results indicated that PICU LoS and hospital LoS, agranulocytosis, hematologic malignancies, blood transfusion, IMV, indwelling urinary catheter, and identified fungal pathogen were independent factors influencing treatment outcomes. Among these factors, identified fungal pathogen was a protective factor, whereas the others were risk factors. The results were visualized in the form of a forest plot (Figure 3).

### 3.4 The results of multivariate logistic regression were utilized to analyze the clinical data and fungal species distributions in the pre-pandemic and post-pandemic groups of children with IFI

**Figure 4:**
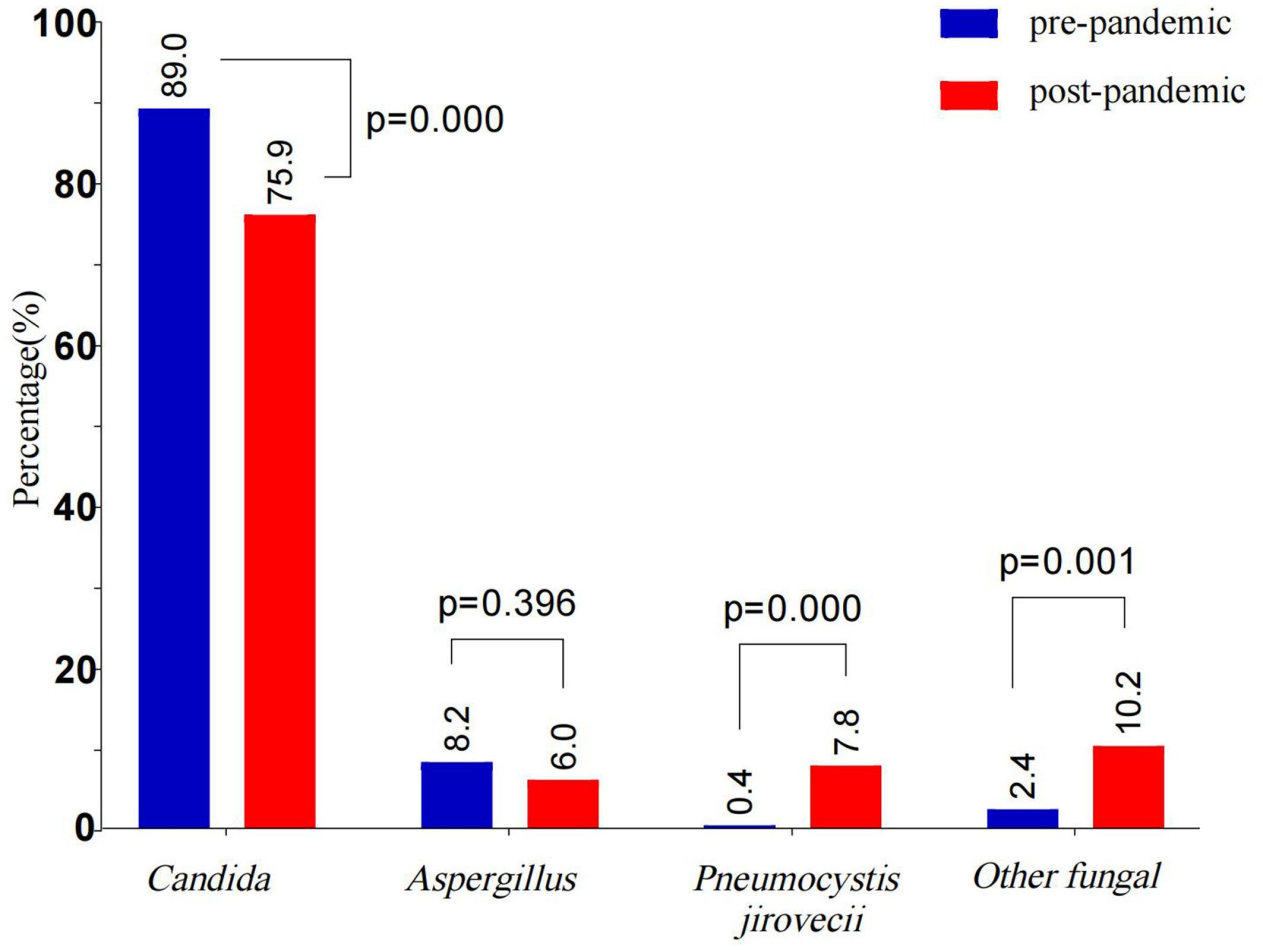
Comparative of fungal species distribution in pre-pandemic and post-pandemic. The bars show the proportion of different fungal species in pre-pandemic and post-pandemic. The intergroup differences were assessed using the Chi-squares test, and *p* < 0.05 indicated statistical significance. Blue bars indicate pre-pandemic, and red bars indicate post-pandemic.

The co-infection rate in the post-pandemic group decreased significantly (*p* < 0.05), whereas the proportion of children with identified fungal pathogens increased compared to the pre-pandemic group. However, the post-pandemic group presented significantly higher rates of agranulocytosis, hematologic malignancies, blood transfusions, IMV, and indwelling urinary catheters compared to the pre-pandemic group (all *p* < 0.05), and a similar trend was noted for the treatment failure rate (*p* < 0.05). Post-pandemic, the proportions of *Candida* and *Aspergillus* decreased, whereas those of *P. jirovecii* and other fungal species increased markedly (*p* < 0.05) (Table 5 and Figure 4).

**Table 5:** Comparison of pre-pandemic and post-pandemic clinical data.

Groups	PICU LoS/day	Hospital LoS/day	Co-infection	IFP	Agranulocytosis	Hematologic malignancies	BT	IMV	IUC	Treatment failure
Prepandemic (421)	19.0 (10.0,37.0)	27.0 (16.0,43.0)	328 (77.9)	255 (60.6)	34 (8.1)	37 (8.8)	232 (55.1)	263 (62.5)	225 (53.4)	193 (45.8)
Postpandemic (244)	18.0 (7.0,35.0)	28.0 (15.0,46.0)	172 (70.5)	166 (68.0)	49 (20.1)	55 (22.5)	155 (63.5)	192 (78.7)	177 (72.5)	136 (55.7)
*Χ*^2^/*Z*	−1.536	−0.194	4.556	3.704	20.384	24.508	4.499	18.805	23.563	6.050
*P*-value	0.125	0.846	0.033	0.054	0.000	0.000	0.034	0.000	0.000	0.014

Data presented are median (IQR) or frequency (%) values. Los, length of stay; IFP, identified fungal pathogen; BT, blood transfusion; IMV, invasive mechanical ventilation; IUC, indwelling urinary catheter.

## 4 Discussion

Invasive fungal infections represent a major global public health challenge, with a disease burden far exceeding the previous estimates. According to recent report, over 6.55 million annual cases of invasive fungal diseases occurred worldwide, leading to 3.8 million deaths, among which 2.5 million deaths were directly attributable to fungal infections, which is a significant increase from the previous estimates of 1.5 million deaths per year (Ikuta et al., 2024). In critically ill patients, especially those with high mortality rates and incurring exorbitant medical costs, critical care medicine has become an urgent issue. In this regard, the present study explored the potential prognostic factors, identifying agranulocytosis, hematological malignancies, blood transfusions, IMV, and indwelling urinary catheters as risk factors, while identified fungal pathogen was revealed as a protective factor. Additionally, a longitudinal comparative analysis was conducted between the pre-pandemic and post-pandemic data of the included patients, which revealed a significant increase in treatment failure rates among children with invasive fungal infections in the PICU during the post-pandemic period, suggesting that public health events may indirectly affect the fungal infection prevention and control system of a hospital.

The epidemiological characteristics of IFI are heterogeneous across regions, populations, and underlying medical conditions. This study revealed that *Candida* species constitute the predominant pathogens of IFI (83.8%), with *Candida albicans* being the dominant one (63.7%). Among non-albicans *Candida* species, the top three species were *Candida parapsilosis* (13.3%), *Candida tropicalis* (11.6%), and *Candida glabrata* (6.2%), and their distribution revealed in this study was consistent with the pathogen composition trend of invasive *Candida* infection in China (Duan et al., 2021). *Aspergillus* was the second most common pathogen (6.3%), and its proportion was consistent with the domestic and international surveillance data (about 6.0–12%) (Montagna et al., 2013; Webb et al., 2018; Zhang and Ren, 2016). The detection of *P. jirovecii* (3.3%) and other fungal species (5.5%) highlights the need to monitor pathogen spectrum changes during immunocompromised states. The mortality associated with IFI often reaches 50% due to complex comorbidities and critical illness in the PICU (Charsizadeh et al., 2018; Ikuta et al., 2024; Li et al., 2024; Montagna et al., 2013). This study defined treatment failure as the involvement of clinical deterioration followed by treatment abandonment and death. Treatment abandonment has become an important cause of death in children in the PICU. In this study, the treatment failure rates for *Candida* species and *P. jirovecii* infections were observed to be 40.8 and 57.1%, respectively, which is consistent with previous reports (Charsizadeh et al., 2018; Ikuta et al., 2024; Li et al., 2024; Montagna et al., 2013). The treatment failure rate for *Aspergillus* spp. and other fungal species is particularly notable: *Aspergillus* spp. presented a treatment failure rate of 71.2%, which is consistent with the reported 70% mortality of pediatric aspergillosis (Hon et al., 2024). The treatment failure rate for other fungal species was as high as 82.6%, with all six cases of mucormycosis ending in death or treatment discontinuation, underscoring the notoriously poor prognosis of mucormycosis. Li et al. (2024) reported 57% in-hospital mortality for PICU patients with IFI, with no survivors among mucormycosis patients. Compared to the other types of IFI, aspergillosis and mucormycosis have more elevated mortality risks in critically ill patients, and this suggests the necessity of prioritized prevention and control measures in intensive care unit (ICU) settings for those patients.

This study also revealed the key factors influencing treatment outcomes in children with IFIs in the PICU through a multivariate logistic regression analysis. The primary findings demonstrated that host immunosuppression status significantly impacted prognosis, with children having agranulocytosis presenting a 3.389-fold increased risk of treatment failure (OR = 3.389), whereas those with hematologic malignancies presented a 3.050-fold greater risk of treatment failure compared to the non-hematologic malignancy patients (OR = 3.050). These immunocompromised states exacerbate fungal susceptibility, delayed diagnosis, and disseminated infections, collectively worsening the prognosis. Faraci et al. (2014) reported that the 90-day cumulative survival rate in children with malignant diseases was 51.2% lower than that in those with non-malignant diseases (75.0% vs. 36.8%, *p* = 0.03), indicating that the host immune status not only influences the clinical progression of fungal infections but may also affect outcomes by altering host-pathogen interactions. The secondary risk factors included blood transfusion, IMV, and indwelling urinary catheters (OR = 2.398, 1.938, and 1.750, respectively). Consistent with these findings, immunosuppressive status, mechanical ventilation, and blood transfusion were identified as the top three risk factors for IFIs in critically ill patients in a meta-analysis by Thomas-Rüddel et al. (2022). The association between massive blood transfusions and fungal infections may stem from the synergistic effects of patients’ immunosuppressed baseline status, medical interventions, and transfusion-related complications (Abad et al., 2020; Thomas-Rüddel et al., 2022). Mechanical ventilation increases the risk of death in pediatric patients with hematologic malignancies by 34-fold and serves as an independent predictor of 28-day mortality in ICU patients with fungemia (Dursun et al., 2020; Paiva et al., 2016). Indwelling urinary catheters may lead to mucosal barrier disruption and biofilm formation, particularly when combined with immunosuppression, prolonged catheterization, and improper handling; these high-risk factors synergistically increase the risk of fungal infections (Thomas-Rüddel et al., 2022). These invasive procedures compromise the physiological barriers, providing entry pathways for opportunistic pathogens. Therefore, adhering to standardized clinical protocols and reinforcing infection control measures in clinical practice are important. Paradoxically, patients in the treatment failure group had shorter PICU LoS and hospital LoS compared to those in the effective treatment group. This was probably due to early death or treatment discontinuation in critical conditions in the former. Although regression analysis revealed significant associations between hospitalization duration and outcomes, the OR values approached 1 (1.022 and 0.953), excluding PICU LoS and hospital LoS from the final risk model.

This study not only identified the independent risk factors affecting the prognosis of IFI but also revealed identified fungal pathogen as a significant protective factor (OR = 0.588, 95% CI [0.362–0.956]). Previous studies have reported that early recognition of bacterial-fungal co-infections contributes to a better implementation of precise antimicrobial therapy for *Pneumocystis* pneumonia, which has been confirmed as a core strategy to reduce mortality and improve outcomes in these patients (Wu et al., 2021). Patients receiving high-dose or prolonged immunosuppressive therapy exhibit significantly increased mortality, whereas early diagnosis and treatment effectively increase survival rates (Riley, 2021). However, caution is warranted to avoid excessive antifungal usage in colonized patients. A recent study published in The Lancet Infectious Diseases revealed that IFI contributes to about 1.5 million deaths globally every year, with the lack of definitive diagnosis and delayed treatment reported as the leading causes of death (Ikuta et al., 2024). Consequently, precise etiological diagnosis has become the cornerstone of improving anti-infective treatment efficacy. Although antifungal therapies have advanced, the continuing use of traditional diagnostic methods frequently causes delays in the treatment of critically ill patients, leading to poor outcomes. In this context, novel diagnostic technologies play a crucial role in terms of clinical value. Molecular approaches, including specific antigen detection, fungal nucleic acid amplification, and metagenomics next-generation sequencing (mNGS), have provided breakthrough directions for early IFI diagnosis. Jia et al. (2023) confirmed that mNGS outperforms conventional microbiological methods in pulmonary aspergillosis diagnosis, offering faster pathogen detection and greater accuracy. However, standardizations of these emerging technologies through large-scale clinical studies and comprehensive evaluations incorporating host immunity, clinical manifestations, and radiological features are nonetheless warranted.

This study enrolled 665 pediatric patients, among whom 438 (65.9%) had fungal-bacterial co-infections, with *Acinetobacter baumannii*, *Pseudomonas aeruginosa*, and *Klebsiella pneumoniae* being the predominant bacteria causing these co-infections. Additionally, 147 patients (22.1%) presented with viral co-infections, with cytomegalovirus (CMV) being the most common virus. The co-infection rates varied across studies, probably due to differences in the population characteristics, immune status, and geographic factors of subjects included in these studies. For example, Zhao et al. (2021) reported bacterial and viral co-detection rates of 40.3 and 17.6%, respectively, in pulmonary fungal infections. Gangneux et al. (2022) reported bacterial ventilator-associated pneumonia in 73% and herpes simplex virus type 1 or CMV infection in 15–10% of all mechanically ventilated patients with IFI. Co-infections significantly worsen prognosis, fungal-CMV co-infection increases mortality, and *Pseudomonas aeruginosa* co-infection is correlated with severe clinical manifestations and poor outcomes (Granchelli et al., 2018; Lécuyer et al., 2022; Wu et al., 2021). Following the COVID-19 pandemic, the incidence and detection rates of common respiratory viruses and *Mycoplasma pneumoniae* in children significantly decreased (Achangwa et al., 2022; Sauteur et al., 2022; Zhu et al., 2023). Containment measures contributed to reduced sepsis-related incidence and mortality in hospital settings (Dutta et al., 2022; Mukherjee et al., 2023). These findings suggest that the control measures adopted during the pandemic effectively suppress the transmission of respiratory pathogens. This study also revealed a notable decline in co-infection rates post-pandemic compared to the pre-pandemic rates (77.9% vs. 70.5%, *p* < 0.05); however, the treatment failure rate for IFI tended to increase post-pandemic (45.8% vs. 55.7%, *p* < 0.05). This may be related to the following factors of the interaction: (1) Changes in high-risk population characteristics: Post-pandemic, there has been an increased proportion of neutropenic patients and children with hematological malignancies, coupled with an increase in invasive procedures. These factors may have offset the potential benefits of reduced mixed infection rates. Nucci et al. (2021) reported that the increasing incidence of candidemia in non-COVID-19 patients is correlated to an increased exposure to risk factors. (2) The fungal pathogen spectrum has undergone significant changes since the pandemic happened, with a decline in the proportions of *Candida* and *Aspergillus* genera, whereas the proportions of *P. jirovecii* and other fungal genera have markedly increased (*p* < 0.05). In the WHO Fungal Priority Pathogens List (FPPL) (Casalini et al., 2024), *P. jirovecii* has been elevated to the highest critical priority tier because of the expansion of its at-risk populations and the associated therapeutic challenges. Other fungal genera, characterized by low clinical incidence, diagnostic difficulties, and high mortality, further exacerbate the clinical management challenges. (3) Limitations of disinfection measures: Alcohol-based hand sanitizers, which were used widely during the pandemic, are effective against bacteria and viruses but exhibit limited efficacy in terms of inactivating fungal spores, which could have increased the risk of fungal colonization in hospital settings (Rutala and Weber, 2008). In summary, this study revealed the dual impact of pandemic control measures on co-infection rates: while these measures reduced the transmission of non-fungal pathogens, they failed to improve clinical outcomes in children with IFI and even worsened the clinical outcomes in certain cases due to pathogen spectrum changes and host factors.

This study employed a multicenter retrospective design to systematically analyze the prognostic factors of invasive fungal infections in the PICU. By including all the eligible cases reported over 5 years, selection bias was effectively controlled. Through multivariable logistic regression models, risk factors and protective factors were comprehensively identified. Furthermore, this study revealed the potential effects of pandemic control measures on IFI outcomes: after the pandemic, co-infection rates decreased, but shifts in fungal species distributions and increased exposure to multiple risk factors for IFI were observed, which led to elevated treatment failure rates. The findings of this study would provide evidence-based insights for optimizing the clinical management of IFI and important information for designing pediatric public health policies. However, the retrospective design of the study inherently limits the complete elimination of confounding factors, and further prospective studies are recommended.

Moreover, the treatment failure rates of invasive fungal infections in PICU patients remain high to date, and special attention should be paid to children with agranulocytosis and hematologic malignancies. Identifying fungal pathogens as early as possible, strictly controlling the indications, and using standardized procedures for blood transfusions, IMV, indwelling catheters, and other invasive interventions could improve the clinical outcomes in these patients.

## Data Availability

The original contributions presented in the study are included in the article/supplementary material, further inquiries can be directed to the corresponding author.
